# A color-coded atlas of the cortical surface anatomy of the equine brain

**DOI:** 10.3389/fvets.2026.1896929

**Published:** 2026-08-19

**Authors:** Ahmad Al Aiyan, Sara Altamimy, Meera Almansoury, Rinsha Balan

**Affiliations:** Department of Veterinary Medicine, College of Agriculture and Veterinary Medicine, United Arab Emirates University, Al Ain, United Arab Emirates

**Keywords:** brain mapping, cerebral sulci and gyri, cerebrum, equine neuroanatomy, neurotopography

## Abstract

**Introduction:**

The horse has a highly gyrencephalic cerebrum; however, detailed references of equine cortical surface anatomy remain limited, and terminology inconsistencies continue to complicate comparative, clinical, and educational applications. This research focused on creating a standardized, high-resolution, color-coded atlas of equine brain sulci and gyri, based on the Nomina Anatomica Veterinaria (NAV).

**Methods:**

Brains from ten neurologically normal horses were examined. Following fixation and dissection, the sulci and gyri of the lateral, dorsal, rostral, and medial surfaces were documented, color-coded, and labeled according to NAV terminology.

**Results:**

The atlas documented a consistent lateral network dominated by the Sylvian fissure with an accessory Sylvian fissure at its origin, a three-limbed ectosylvian sulcus that subdivides the ectosylvian gyrus, a continuous suprasylvian sulcus, and a distinct oblique sulcus. Dorsally, the coronal, cruciate, and ansate sulci are complemented by a well-developed marginal complex (marginal, ectomarginal, and endomarginal sulci). Medially, the genual region is resolved into genual, endogenual, and ectogenual sulci; the callosal and cingulate sulci run in parallel; and the splenial complex (splenial, endosplenial, and ectosplenial) is consistently identifiable alongside the medial rhinal sulcus. Across specimens, major sulci and gyri were observed, with variation limited primarily to differences in sulcal depth and branching patterns.

**Conclusion:**

This standardized, color-coded surface atlas provides a detailed reference for equine cortical anatomy, facilitates cross-species comparison, and offers a practical anatomical resource for neuroimaging interpretation, anatomical orientation, and veterinary education. The atlas serves as a specimen-based resource that may provide an anatomical framework for future morphometric, developmental, and functional investigations.

## 1 Introduction

The horse is one of the largest domestic mammals, with a highly gyrencephalic brain that has long been of interest in comparative and translational neuroscience.

The distinct cortical folding observed in the horse’s brain, along with its cognitive and behavioral abilities such as learning and memory, makes it a valuable large-animal model for neuroanatomical and clinical research (1, 2).

Despite this, the detailed neuroanatomy of the equine cerebrum remains underexplored. Much of the information in textbooks is derived from century-old descriptions or single specimens (1), leaving a gap in systematic, high-resolution mapping of equine sulci and gyri.

Early studies attempted to describe equine cerebral fissures and gyri but produced inconsistent terminology and incomplete maps.

Schmidt (3) utilized MRI to document equine surface morphology but did not provide a detailed, clear sulcal description, whereas Johnson et al. (2) generated a stereotaxic atlas focusing on volumetric and stereotactic data rather than surface folding.

Lang et al. (4) emphasized that the equine cortex is highly convoluted, with secondary sulci and considerable variability between individuals, and noted that the terms sulcus and fissure are often used incorrectly, complicating communication among anatomists and clinicians.

Recent advances in artificial intelligence (AI) have expanded the applications of anatomical datasets in both veterinary education and diagnostic imaging. High-quality, standardized anatomical atlases provide essential reference material for the development of AI-assisted image interpretation, automated anatomical segmentation, and digital educational resources. However, the effectiveness of AI-based approaches depends on the availability of accurate, consistently annotated anatomical references, highlighting the continued importance of detailed specimen-based atlases in veterinary anatomy (5–7).

In addition to supporting anatomical research, detailed animal anatomical models play an important role in veterinary and biomedical education, surgical training, and the development of experimental models for medical research (8, 9). High-quality anatomical specimens remain fundamental to veterinary anatomy education, and advances in specimen preservation have further improved their long-term educational value (10).

Comparative work has further revealed how equine cortical folding differs from that of other domestic species. The horse exhibits continuous presylvian, coronal, and suprasylvian sulci, in contrast to the fragmented sulci of ruminants (11, 12). Among bovids, the presylvian sulcus often terminates blindly without forming a distinct prorean gyrus (13), whereas in the horse, this gyrus is consistently present.

Studies of camels have demonstrated a unique but less ramified sulcal pattern (14, 15). Further comparative work detailing the intricate neurovascular architecture in camels (16–19) also highlights the species-specific differences in cerebral organization. These interspecies comparisons highlight the evolutionary divergence of cortical topography and the importance of species-specific atlases.

Despite previous descriptions of the equine cerebrum, currently available resources remain limited by their emphasis on MRI visualization, stereotaxic mapping, historical descriptions, or inconsistencies in anatomical terminology. A modern specimen-based atlas that comprehensively documents the external cortical surface using standardized NAV nomenclature and consistent multiview illustrations has remained unavailable.

Accordingly, the objective of the present study was to develop a comprehensive, color-coded atlas of the external cortical anatomy of the equine brain based on multiple adult specimens and standardized Nomina Anatomica Veterinaria (NAV) terminology. The study systematically documented the sulci and gyri visible on the lateral, dorsal, rostral, and medial surfaces, including accessory and subdivided features reported in the literature, and integrated them into a unified anatomical framework.

The resulting atlas is intended to provide a standardized anatomical reference for comparative neuroanatomy, veterinary education, and future anatomical investigations of the equine brain.

## 2 Materials and methods

Adult equine brains were obtained opportunistically from ten mixed-breed horses that were euthanized for reasons unrelated to this study and had no history of neurological disease. The study included 4 males and 6 females ranging from approximately 7–10 years of age. The heads were collected from several veterinary clinics in Al Ain, Abu Dhabi.

All specimens were handled and transported to the Department of Veterinary Medicine at the United Arab Emirates University under institutional biosecurity guidelines.

The study involved collecting post-mortem specimens from animals already deceased for unrelated clinical reasons. Consequently, under United Arab Emirates University guidelines, ethics approval from the IACUC was not needed.

To examine the exterior architecture of the cerebrum, the skin was first removed from each head, and the dorsal cranium was opened using a saw.

The intact heads were submerged in 10% neutral buffered formaldehyde for one month at 5 °C to ensure thorough fixation of the neural tissues.

Following fixation, the skulls were carefully opened with surgical instruments, the brains were extracted, and the meninges were removed to expose the cortical surfaces.

Each cerebrum was divided along the longitudinal fissure to obtain two hemispheres. The gyri and sulci on the lateral, dorsal, rostral, and medial aspects were inspected visually and documented as high-resolution digital photomacrographs using a Sony a7R II digital camera. All specimens were photographed under consistent lighting conditions with the camera positioned at approximately the same distance from each specimen to maintain comparable image orientation and scale. Particular attention was given to the identification of major cortical landmarks and the assessment of their consistency across specimens and between hemispheres.

To enhance the consistency of anatomical identification, the sulci and gyri were independently identified by the participating veterinary anatomists. Following the independent evaluations, all observations were reviewed collectively, and any discrepancies in anatomical identification or nomenclature were resolved by consensus using the Nomina Anatomica Veterinaria (NAV) as the reference standard.

The image files were subsequently processed in Adobe Photoshop (version 25.5), where minor adjustments to brightness and contrast were made as needed to optimize image quality without altering anatomical features. The sulci and gyri were color-coded and labeled according to the NAV.

## 3 Results

This study examines the external architecture of the horse brain, emphasizing its gross anatomy as viewed from the lateral, dorsal, rostral, and medial aspects. Across all examined specimens, the principal sulci and gyri were consistently identified, whereas variation was limited primarily to differences in sulcal depth, branching patterns, and segment length.

### 3.1 Sulci of the horse brain

The cortical surface of the horse exhibits a markedly convoluted pattern characterized by numerous deep, continuous, and variably branched sulci.

Inspection of the cerebral hemispheres revealed a complex and distinctive network of sulci that shape the exterior surface and provide consistent landmarks for cortical topography.

#### 3.1.1 Lateral sulci

##### 3.1.1.1 Sylvian fissure

**Figure 1:**
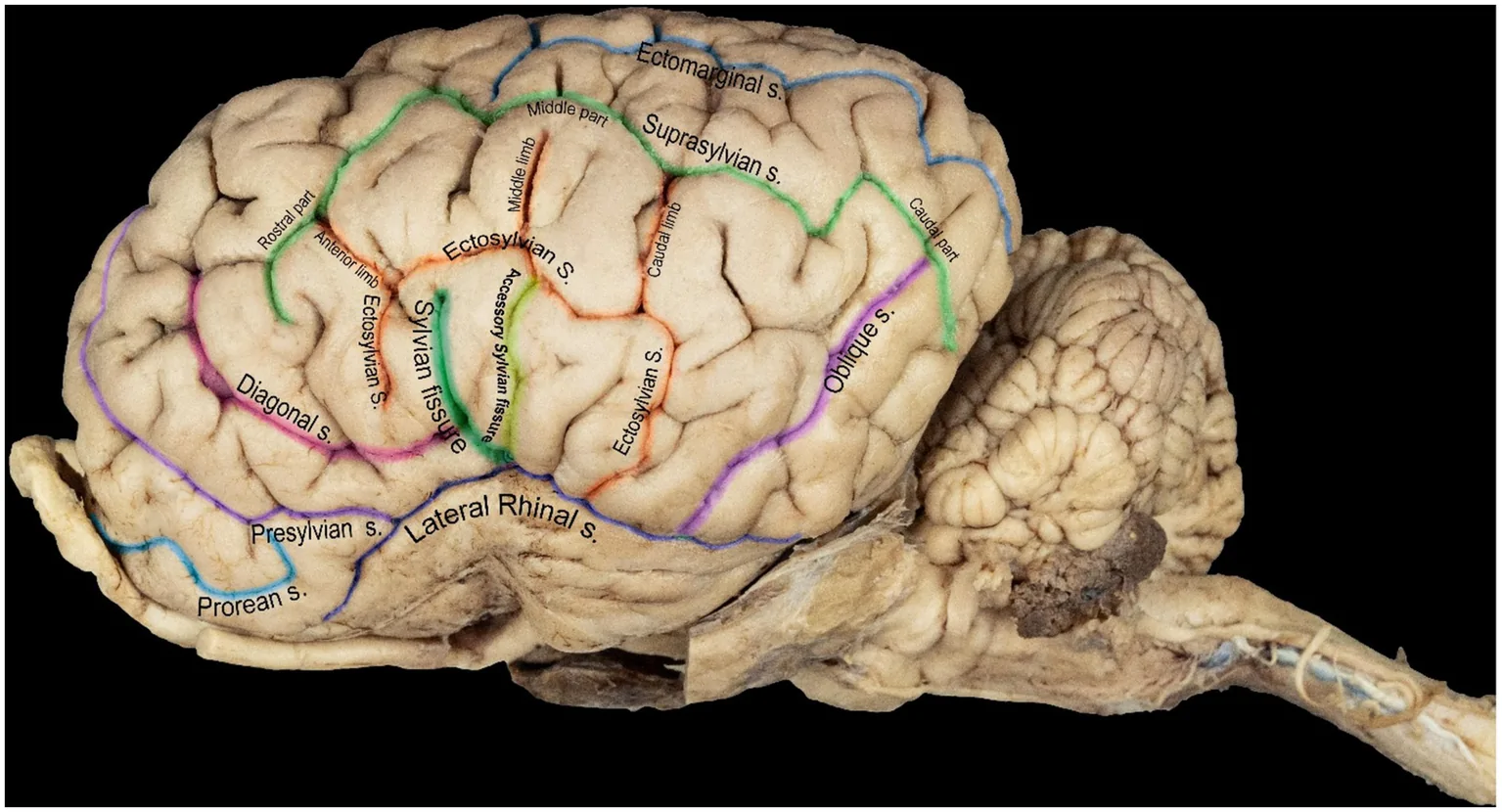
Lateral view of the left cerebral hemisphere of the horse showing the sulci color-coded and labeled according to the Nomina Anatomica Veterinaria (NAV).

The Sylvian fissure is clearly visible from the lateral or ventrolateral view of the horse hemisphere. It begins near the lateral rhinal sulcus and extends slightly in a rostro-dorsal direction (Figure 1).

**Figure 2:**
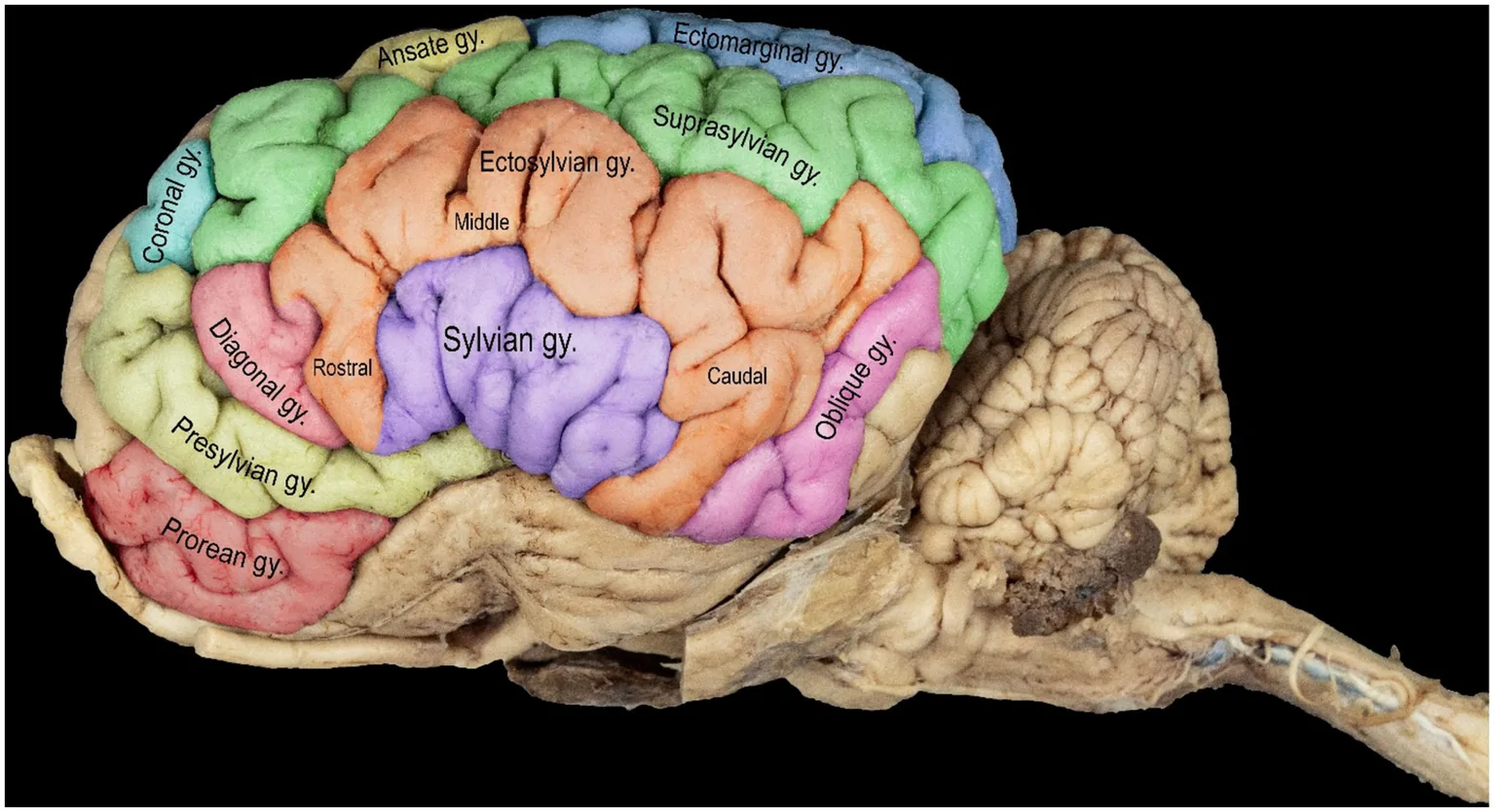
Lateral view of the left cerebral hemisphere of the horse showing the gyri color-coded and labeled according to the Nomina Anatomica Veterinaria (NAV).

From the origin of the Sylvian fissure, an additional sulcus, referred to as the accessory Sylvian fissure (Figure 1), is present and surrounded by the Sylvian gyrus (Figure 2).

##### 3.1.1.2 Lateral rhinal sulcus

The lateral rhinal sulcus forms a continuous and well-defined landmark along the ventrolateral aspect of the hemisphere. It originates rostrally near the prorean and presylvian regions and extends caudally in a nearly straight rostrocaudal direction toward the temporal pole (Figure 1).

The sulcus runs parallel to the ventral margin of the hemisphere and demarcates the boundary between the paleocortical structures of the piriform lobe and the overlying neocortical regions.

Throughout its course, it maintains a uniform depth and clear outline, making it one of the most stable and easily identifiable sulci of the horse brain (Figure 1).

##### 3.1.1.3 Prorean sulcus

The prorean sulcus is a shallow but consistent landmark located on the rostral aspect of the lateral hemisphere. It originates near the frontal pole, just rostral to the presylvian sulcus, and extends toward the olfactory bulb region (Figure 1).

The sulcus is relatively short and slightly curved. Its orientation delineates the dorsal and ventral portions of the prorean gyrus, which forms the most rostral cortical surface of the horse brain. In relation to surrounding structures, the prorean sulcus lies rostral to the presylvian sulcus, dorsal to the olfactory tract, and lateral to the lateral rhinal sulcus (Figure 1).

##### 3.1.1.4 Presylvian sulcus

The presylvian sulcus is a distinct groove located rostral to the Sylvian fissure on the lateral aspect of the hemisphere.

It begins ventrally near the lateral rhinal sulcus and ascends dorsorostrally toward the frontal pole. The sulcus is moderately deep and relatively straight, maintaining a consistent course without major branching.

It forms the caudal boundary of the prorean region and runs parallel to the diagonal sulcus dorsally. In relation to surrounding landmarks, the presylvian sulcus lies rostral to the Sylvian fissure and ventral to the diagonal sulcus (Figure 1).

##### 3.1.1.5 Diagonal sulcus

The diagonal sulcus is a clearly identifiable groove located on the lateral aspect of the hemisphere. It originates from the Sylvian fissure and courses obliquely in a rostrodorsal direction.

It defines the ventral boundary of the diagonal gyrus and runs dorsally parallel to the presylvian sulcus (Figures 1, 2). Rostral to its extent, the diagonal sulcus approaches the presylvian sulcus, while caudally it terminates short of the Sylvian fissure (Figure 1).

##### 3.1.1.6 Ectosylvian sulcus

The ectosylvian sulcus is a prominent and consistently developed groove situated dorsal to the Sylvian fissure.

It originates rostrally near the diagonal sulcus and extends dorsocaudally, maintaining a close parallel relation to the suprasylvian sulcus dorsally. The sulcus is deep and elongated and typically gives rise to three limbs—anterior, middle, and caudal (Figure 1).

These subdivisions delineate the ectosylvian gyrus into corresponding rostral, middle, and caudal parts (Figure 2).

The anterior limb converges rostrally toward the rostral part of the suprasylvian sulcus, the middle limb runs upward toward the suprasylvian sulcus, and the caudal limb curves slightly dorsocaudally toward its caudal part.

Throughout its course, the ectosylvian sulcus retains a uniform depth and well-defined curvature, making it one of the most characteristic lateral sulci of the equine hemisphere.

##### 3.1.1.7 Suprasylvian sulcus

The suprasylvian sulcus is among the most prominent and extensive grooves on the lateral surface of the equine hemisphere. It begins rostrally at the rostrodorsal aspect of the hemisphere and extends caudally in a curved longitudinal direction toward the occipital region (Figure 1).

The sulcus is deep and continuous along its course and is generally divided into three portions—rostral, middle, and caudal (Figure 1). The rostral segment lies rostrodorsal to the rostral ectosylvian limb, the middle segment runs parallel to the ectosylvian sulcus, and the caudal segment curves caudally toward the oblique sulcus (Figure 1).

This subdivision delineates the suprasylvian gyrus into distinct regions. The sulcus maintains a generally uniform depth, although minor variations in branching and ramification were observed among specimens. It shows consistent relationships ventrally with the ectosylvian sulcus and dorsally with the marginal and ectomarginal complex, making it a reliable and defining landmark of the equine lateral cortex (Figure 1).

##### 3.1.1.8 Oblique sulcus

The oblique sulcus is a distinct groove situated at the caudal portion of the lateral hemisphere near the junction of the temporal and occipital regions. It originates ventrally from the lateral rhinal sulcus, close to the caudal end of the ectosylvian sulcus, and extends dorsocaudally in an oblique direction.

The oblique sulcus defines the ventrocaudal boundary of the oblique gyrus. Its consistent orientation and termination make it a useful landmark for identifying the transition between the temporal and occipital cortical fields in the horse brain (Figure 1).

#### 3.1.2 Dorsal sulci

##### 3.1.2.1 Coronal sulcus

**Figure 3:**
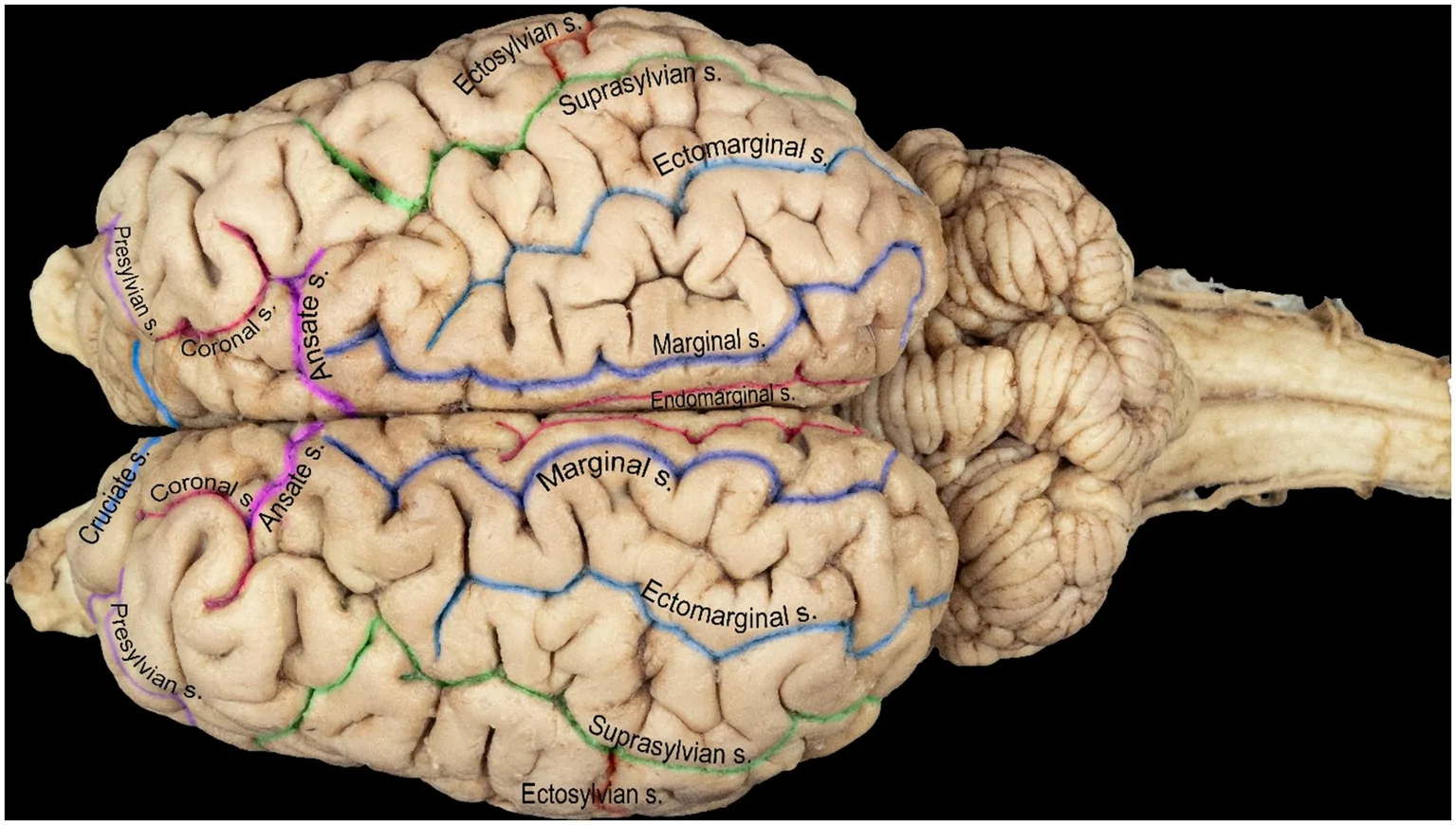
Dorsal view of the brain of the horse showing the cerebral sulci color-coded and labeled according to the Nomina Anatomica Veterinaria (NAV).

**Figure 4:**
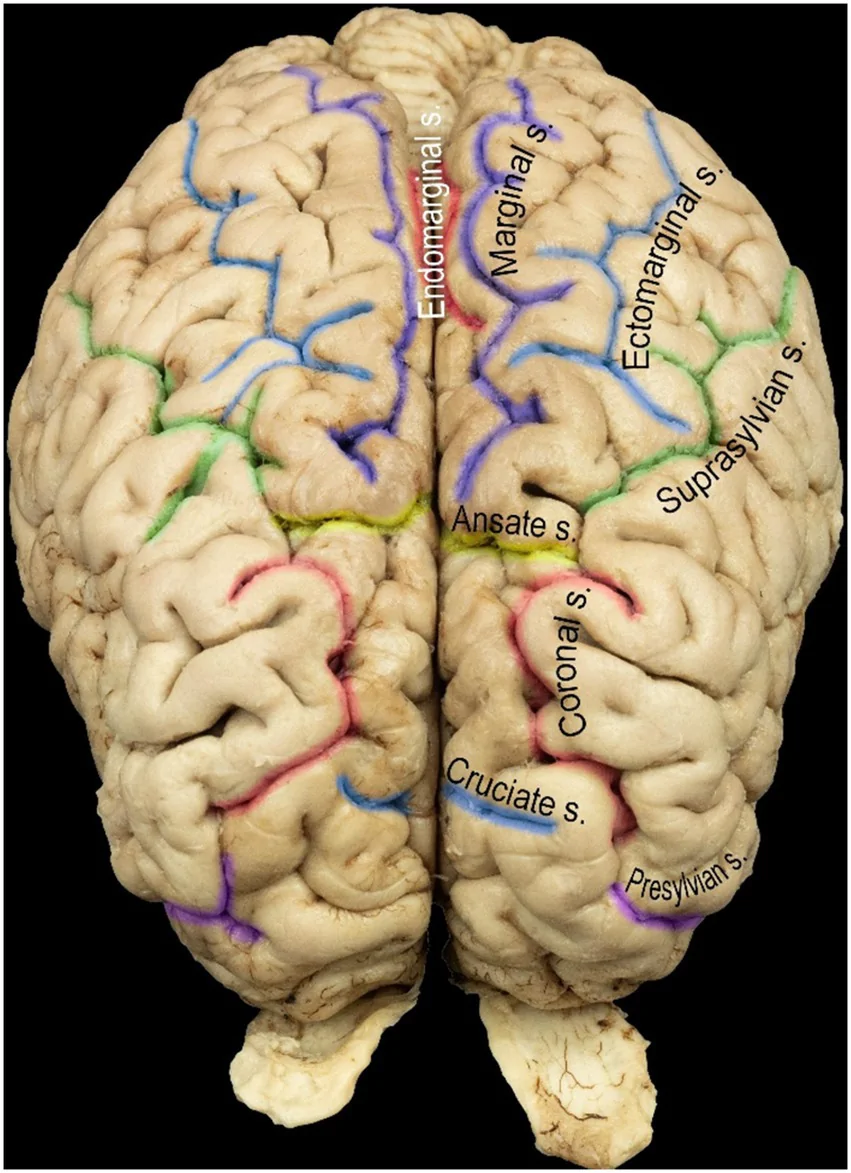
Rostral view of the brain of the horse showing the cerebral sulci color-coded and labeled according to the Nomina Anatomica Veterinaria (NAV).

The coronal sulcus is a well-defined groove located on the rostrodorsal surface of the equine cerebral hemisphere. It originates medially near the longitudinal fissure and extends laterally across the frontal region in a transverse orientation (Figures 3, 4).

The sulcus is moderately deep, short, and follows a nearly curved course along its trajectory (Figures 3, 4).

In most hemispheres, the sulcus appeared continuous, although minor variations in length were noted between the left and right sides. The coronal sulcus consistently delineates the rostral dorsal surface and serves as a reliable landmark separating the anterior frontal field from the cruciate region (Figures 3, 4).

##### 3.1.2.2 Cruciate sulcus

The cruciate sulcus is a short but distinct transverse groove situated on the rostrodorsal aspect of the frontal region. It originates medially at the longitudinal fissure and extends laterally for a relatively short distance before terminating within the cortical surface.

The sulcus is moderately deep and straight, with a clear orientation perpendicular to the longitudinal axis of the brain. In the examined specimens, the cruciate sulcus was consistently present on both hemispheres, although minor asymmetries were noted in its length and lateral extension (Figure 4).

##### 3.1.2.3 Ansate sulcus

The ansate sulcus is a dorsal groove that arises medially from the longitudinal fissure, just caudal to the cruciate sulcus. From its medial origin, it extends laterally, running obliquely across the dorsal surface of the cerebral hemisphere (Figures 3, 4).

The sulcus is moderately deep and broader than the cruciate sulcus.

Rostral to the ansate sulcus lie the cruciate and coronal regions, whereas caudally it borders the dorsal field of the marginal gyrus. In most specimens, the sulcus appeared continuous, although slight variations in curvature and lateral extension were observed (Figures 3, 4).

##### 3.1.2.4 Marginal sulcus

The marginal sulcus is one of the longest and most visible grooves on the dorsal surface of the equine hemisphere (Figures 3, 4).

It originates rostrally near the medial aspect of the ansate sulcus, close to the longitudinal fissure, and extends caudally toward the occipital pole (Figures 3, 4).

The sulcus is consistently deep, straight in its medial portion, and gently curved laterally as it courses caudally.

It runs parallel to the longitudinal fissure and defines the dorsal border of the marginal gyrus. In most hemispheres, the sulcus was uninterrupted, although occasional short accessory branches or slight asymmetry in length were observed (Figures 3, 4).

##### 3.1.2.5 Ectomarginal sulcus

The ectomarginal sulcus runs parallel and lateral to the marginal sulcus. It originates rostrally at a point slightly lateral to the medial ansate region and courses caudally in close correspondence with the marginal sulcus, extending toward the occipital cortex (Figures 3, 4).

**Figure 5:**
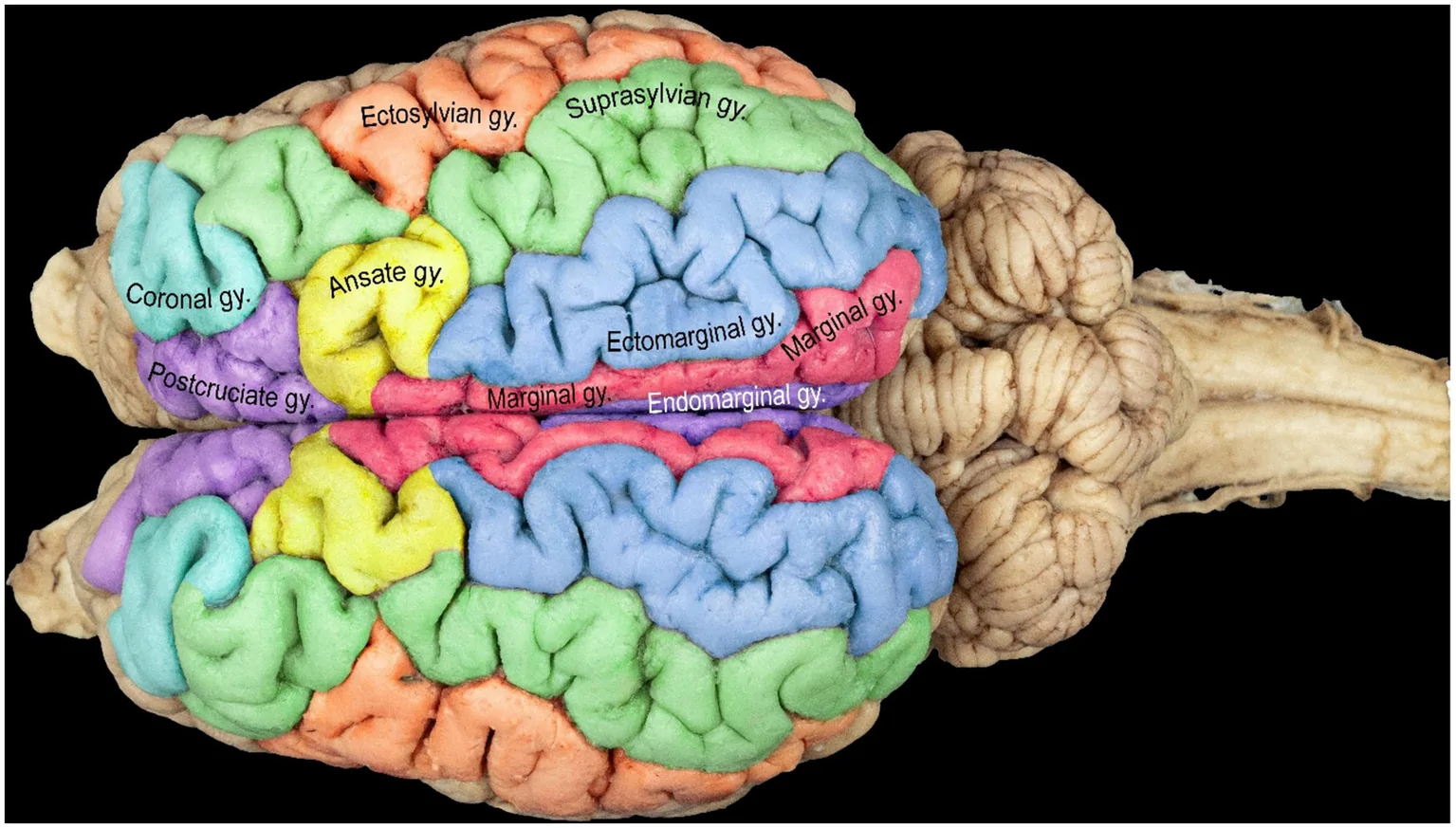
Dorsal view of the horse cerebral hemispheres showing the gyri color-coded and labeled according to the Nomina Anatomica Veterinaria (NAV).

The ectomarginal sulcus defines the lateral boundary of the ectomarginal gyrus, lying between the marginal sulcus medially and the ectomarginal sulcus laterally (Figures 2, 5). Its course is mostly straight, although minor side branches were noted in several specimens (Figures 3, 4).

##### 3.1.2.6 Endomarginal sulcus

The endomarginal sulcus is a finer, less distinct groove situated medial to the marginal sulcus.

It arises close to the longitudinal fissure and extends caudally in a nearly parallel course to the marginal sulcus. The sulcus is shallow and shorter than both the marginal and ectomarginal sulci and is rarely interrupted along its path in some specimens.

**Figure 6:**
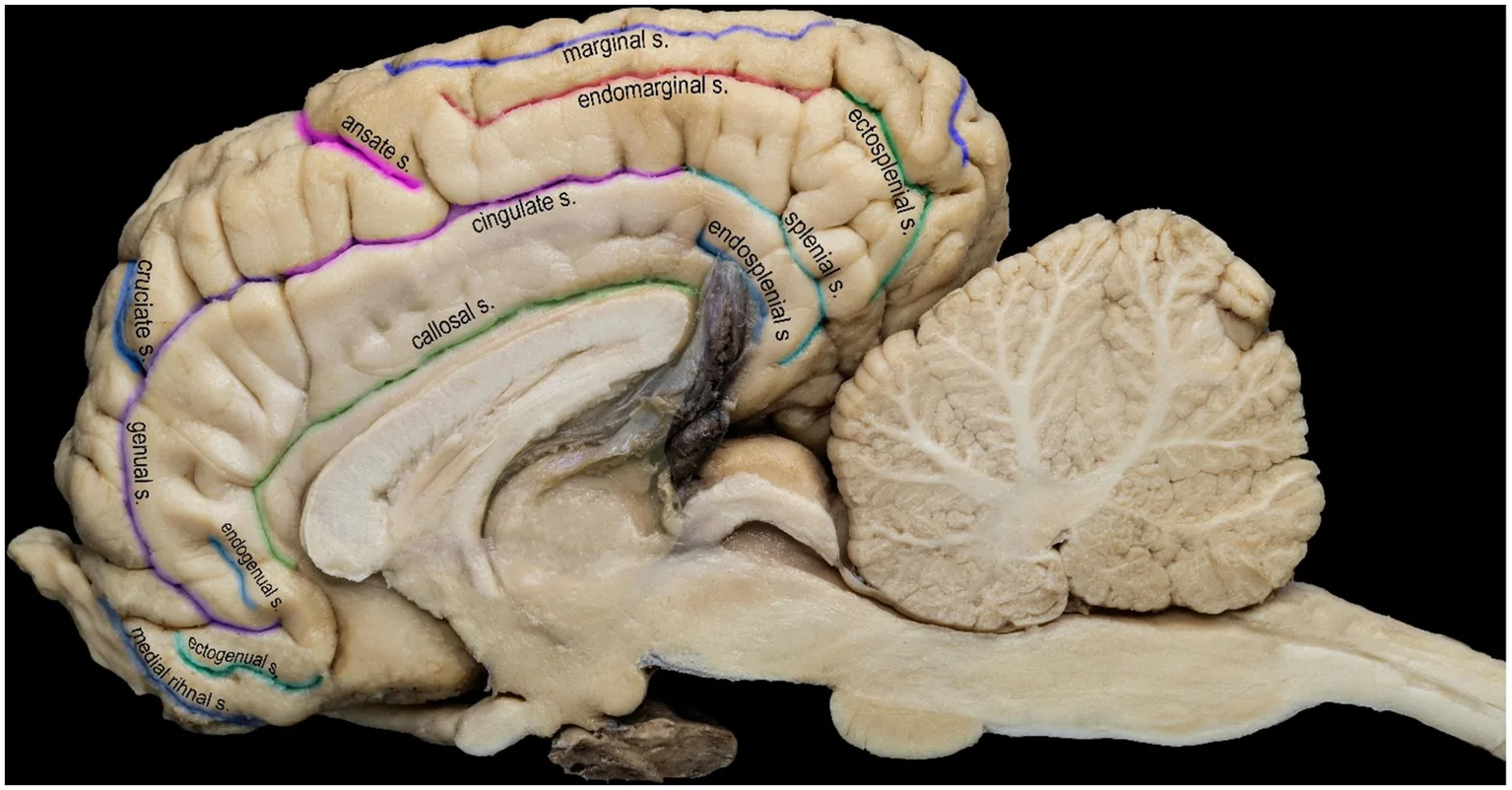
Medial view of the right hemisphere of the horse showing the cerebral sulci color-coded and labeled according to the Nomina Anatomica Veterinaria (NAV).

Despite its variability, the endomarginal sulcus was identifiable in most examined hemispheres (Figures 3, 4 and 6).

#### 3.1.3 Medial sulci

##### 3.1.3.1 Genual sulcus

The genual sulcus is a distinct medial landmark located rostral to the genu of the corpus callosum (Figure 6).

It originates dorsally near the longitudinal fissure and extends ventrorostrally toward the rostral commissural region. The sulcus is moderately deep and maintains a curved course that follows the outline of the callosal genu (Figure 6).

Dorsally, it meets the medial end of the cruciate sulcus, while ventrally it approaches the prorean gyrus. The genual sulcus forms the dorsal boundary of the genual gyrus and is closely associated with the adjacent endogenual and ectogenual sulci (Figure 6).

##### 3.1.3.2 Endogenual sulcus

The endogenual sulcus is a short and shallow groove situated medial to the genual sulcus. It arises dorsally near the rostral segment of the callosal sulcus and extends ventrorostrally in a short, slightly curved course that parallels the genu of the corpus callosum. The sulcus often appears as a series of short indentations rather than a single uninterrupted depression. In most specimens, it was evident on both hemispheres, although minor asymmetries in depth and continuity were noted (Figure 6).

##### 3.1.3.3 Ectogenual sulcus

The ectogenual sulcus is located ventral to the genual sulcus. It originates from the rostral medial surface near the medial rhinal sulcus and extends caudally.

The sulcus is moderately deep and more elongated than the endogenual sulcus, with a slightly oblique orientation (Figure 6).

##### 3.1.3.4 Callosal sulcus

The callosal sulcus is a long and continuous groove that closely follows the dorsal margin of the corpus callosum (Figure 6).

It originates rostrally near the genu and extends caudally in a gently curved course, terminating at the level of the splenium.

The sulcus is moderately deep and maintains a smooth contour that mirrors the curvature of the callosal body.

Dorsally, it is bordered by the cingulate gyrus and runs parallel to the cingulate sulcus. The sulcus was consistently present and symmetrical in all examined hemispheres, without major interruptions or accessory branches (Figure 6).

##### 3.1.3.5 Cingulate sulcus

The cingulate sulcus is a prominent and elongated groove situated dorsal to the callosal sulcus, extending parallel to it along the medial surface (Figure 6).

It originates rostrally near the genual region and courses caudally toward the parietal lobe, where it approaches the splenial area.

The sulcus defines the dorsal boundary of the cingulate gyrus, separating it from the endomarginal gyrus on the dorsal surface. In most specimens, the cingulate sulcus maintained a uniform depth and orientation, with only minor hemispheric asymmetries in length (Figure 6).

##### 3.1.3.6 Splenial sulcus

The splenial sulcus is a distinct medial groove located caudal to the cingulate sulcus, in close relation to the splenium of the corpus callosum.

It originates dorsally near the longitudinal fissure and extends ventrocaudally toward the caudal pole of the hemisphere. The sulcus is moderately deep, shorter than the cingulate or callosal sulci, and slightly curved along its course (Figure 6). Dorsally, it approaches the marginal complex and the splenial gyrus. The sulcus was consistently present in all hemispheres.

##### 3.1.3.7 Endosplenial sulcus

The endosplenial sulcus is a finer groove located medial and parallel to the splenial sulcus. It begins near the caudal aspect of the callosal sulcus and runs caudally along the medial wall of the cerebral hemisphere (Figure 6).

The sulcus is shallow and less continuous than the splenial sulcus, often appearing as a series of short indentations rather than a single uninterrupted depression.

##### 3.1.3.8 Ectosplenial sulcus

The ectosplenial sulcus lies lateral and dorsal to the splenial sulcus, forming the outer boundary of the splenial gyrus.

It arises dorsally near the caudal extension of the endomarginal sulcus and courses ventrocaudally, converging toward the caudal pole of the equine cerebral hemisphere. The sulcus is moderately deep and more variable in length than the splenial sulcus. Its oblique orientation distinguishes it from the parallel alignment of the splenial sulcus (Figure 6). By outlining the lateral limit of the splenial gyrus, the ectosplenial sulcus completes the tripartite sulcal system of the caudal medial cortex.

##### 3.1.3.9 Medial rhinal sulcus

The medial rhinal sulcus is a long, continuous groove situated along the ventral aspect of the medial hemisphere (Figure 6).

It originates rostrally near the olfactory bulb and extends caudally in a gently curved course.

The sulcus is moderately deep, more pronounced rostrally, and gradually becomes shallower caudally (Figure 6).

### 3.2 Gyri of the horse brain

#### 3.2.1 Lateral gyri

##### 3.2.1.1 Prorean gyrus

**Figure 7:**
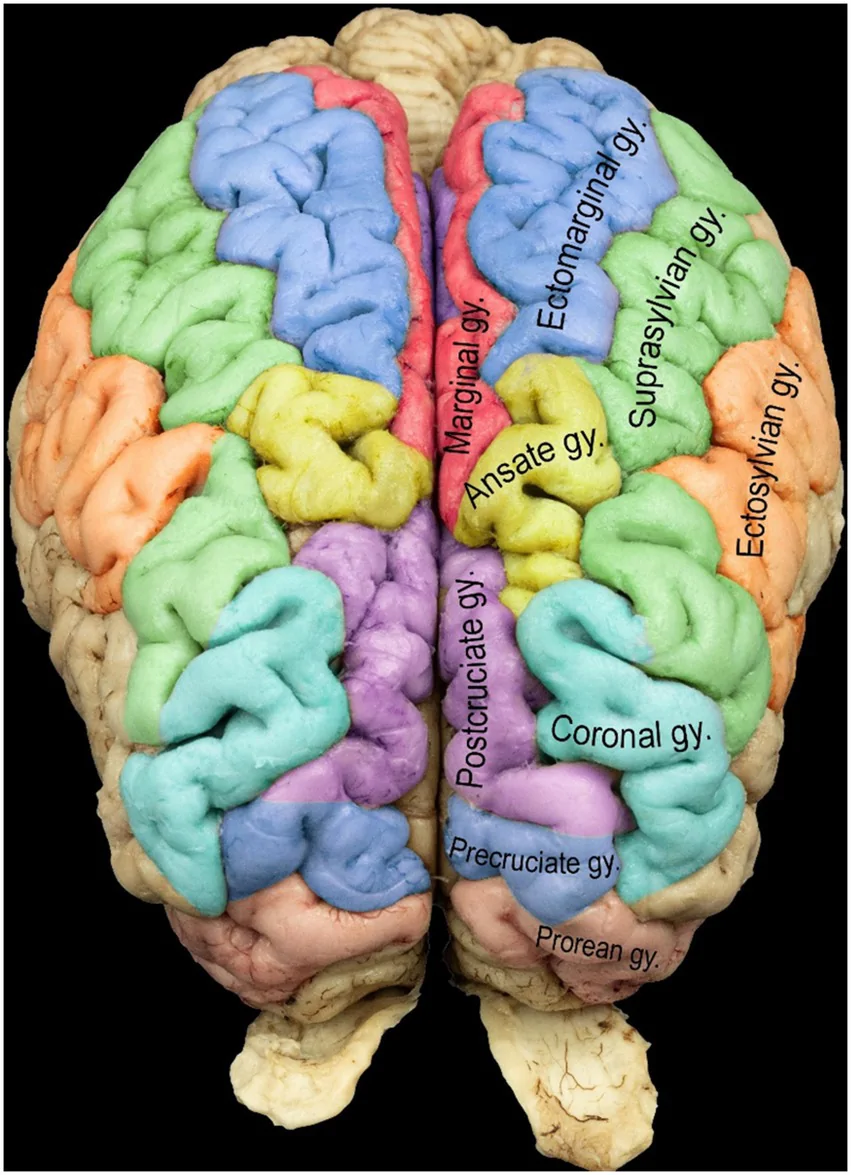
Rostral view of the horse cerebral hemispheres showing the gyri color-coded and labeled according to the Nomina Anatomica Veterinaria (NAV).

The prorean gyrus forms the most rostral portion of the lateral hemisphere. It is situated immediately caudal to the olfactory bulb region and is bounded dorsally by the presylvian sulcus and the presylvian gyrus. Its surface is relatively smooth and convex, occupying the rostral pole of the frontal cortex (Figures 2, 7).

##### 3.2.1.2 Presylvian gyrus

The presylvian gyrus lies caudal to the prorean gyrus and rostral to the diagonal gyrus. It is bounded ventrally by the presylvian sulcus and the lateral rhinal sulcus, and dorsally by the diagonal sulcus. This gyrus is elongated in the rostrocaudal direction (Figure 2).

##### 3.2.1.3 Diagonal gyrus

The diagonal gyrus is positioned caudal to the presylvian gyrus and rostral to the Sylvian and ectosylvian gyri. It is bounded ventrally by the diagonal sulcus and the presylvian gyrus. Its shape is wedge-like, with a pointed caudal end, and it serves as a distinct transitional zone between the rostral and lateral cortical regions (Figure 2).

##### 3.2.1.4 Sylvian gyrus

The Sylvian gyrus is a prominent structure located around the Sylvian fissure.

It is consistently broad, with a convex surface, and occupies the ventrolateral portion of the cortical field (Figure 2).

##### 3.2.1.5 Ectosylvian gyrus

The ectosylvian gyrus extends dorsally to the Sylvian gyrus and is outlined ventrally by the ectosylvian sulcus, dorsally by the suprasylvian sulcus, rostrally by the diagonal sulcus, and caudally by the oblique sulcus. It is divided into rostral, middle, and caudal portions corresponding to the three limbs of the ectosylvian sulcus (Figure 2). The gyrus is elongated, relatively broad, and shows clear separation into subdivisions (Figure 2).

##### 3.2.1.6 Suprasylvian gyrus

The suprasylvian gyrus lies dorsal to the ectosylvian gyrus and is bounded ventrally by the suprasylvian sulcus. It extends longitudinally across much of the lateral hemisphere, from the coronal gyrus rostrally to the caudal end of the occipital cortex. Its dorsal contour is relatively smooth and continuous (Figures 2, 5).

##### 3.2.1.7 Ectomarginal gyrus

The ectomarginal gyrus is located dorsally above the suprasylvian gyrus and below the marginal gyrus.

It is narrow and elongated, with a linear rostrocaudal orientation. It is consistently bounded by the ectomarginal sulcus ventrally and the marginal sulcus dorsally (Figures 2, 5).

##### 3.2.1.8 Oblique gyrus

The oblique gyrus occupies the caudal lateral surface, bounded rostrally by the oblique sulcus and the ectosylvian gyrus, and dorsally by the caudal segment of the suprasylvian sulcus and gyrus.

It has an oblique course and defines the transition between the temporal and occipital regions (Figure 2).

#### 3.2.2 Dorsal gyri

##### 3.2.2.1 Marginal gyrus

The marginal gyrus is the most prominent longitudinal gyrus in the dorsal equine hemisphere.

It extends rostrocaudally, parallel to the longitudinal fissure, and is bounded laterally by the marginal sulcus and medially by the endomarginal sulcus and the hemispheric midline. The gyrus is elongated, broad in its caudal part, and tapers slightly toward the occipital pole of the hemisphere (Figures 5, 7). Its surface is convex and smooth, with minor asymmetries in breadth between hemispheres.

##### 3.2.2.2 Ectomarginal gyrus

The ectomarginal gyrus lies lateral to the marginal gyrus, separated from it by the marginal sulcus, and bounded laterally by the ectomarginal sulcus. It runs parallel to the marginal gyrus, forming a broad and elongated band. The gyrus is relatively uniform in width but broader than the marginal gyrus (Figures 5, 7).

##### 3.2.2.3 Endomarginal gyrus

**Figure 8:**
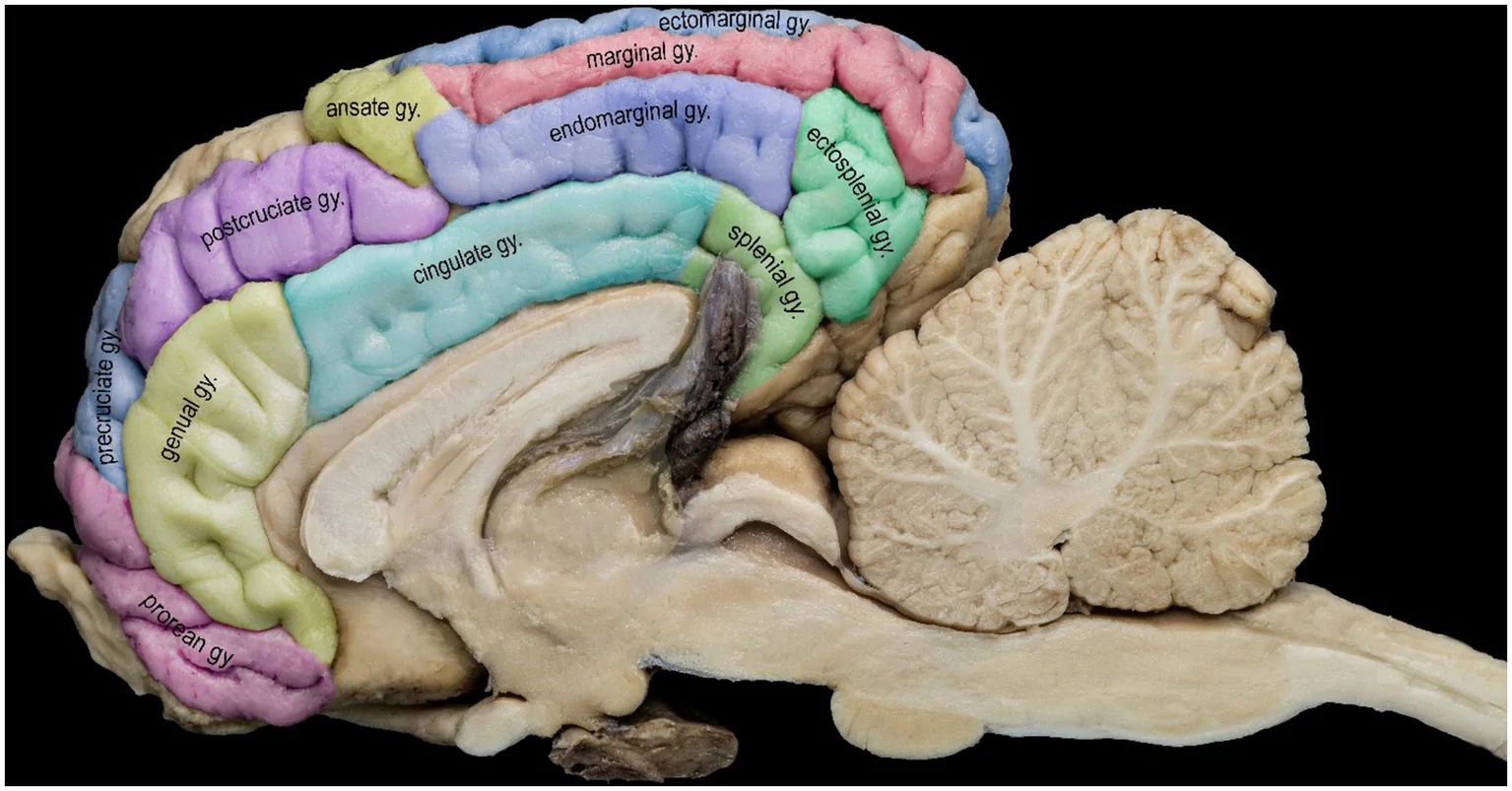
Medial view of the right cerebral hemisphere of the horse showing the gyri color-coded and labeled according to the Nomina Anatomica Veterinaria (NAV).

The endomarginal gyrus is situated ventromedial to the marginal gyrus, separated from it by the endomarginal sulcus. It follows a parallel longitudinal course, extending from the mid-rostral to the caudal hemisphere. This gyrus is narrower and less elevated in the dorsal view compared to the marginal and ectomarginal gyri and is more visible in the medial view of the hemisphere (Figures 5, 8).

##### 3.2.2.4 Ansate gyrus

The ansate gyrus is positioned rostrally on the dorsal surface, bounded rostrally by the coronal and postcruciate gyri and caudally by the marginal and ectomarginal gyri. It is relatively broad, forming a transverse arch across the rostral hemisphere. In most specimens, the gyrus was continuous and convex, although minor hemispheric asymmetry was observed in width (Figures 5, 7 and 8).

##### 3.2.2.5 Coronal gyrus

The coronal gyrus lies at the rostral dorsal aspect of the hemisphere. It is bounded dorsomedially by the coronal sulcus and the postcruciate gyrus. In the dorsal view, it appears as a rostral continuation of the suprasylvian gyrus (Figures 5, 7 and 8).

Its surface is broad, convex, and continuous with the prefrontal region rostrally.

##### 3.2.2.6 Precruciate gyrus

The precruciate gyrus occupies the dorsal surface rostral to the cruciate sulcus.

It is bounded caudally by the cruciate sulcus, rostrally by the prorean gyrus, and laterally by the coronal gyrus.

The gyrus is moderately broad and consistently present in all hemispheres (Figures 5, 7 and 8).

##### 3.2.2.7 Postcruciate gyrus

The postcruciate gyrus is visible in dorsal, rostral, and medial views (Figures 5, 7 and 8). It lies caudal to the cruciate sulcus and rostral to the ansate sulcus and gyrus. It is bounded laterally by the coronal gyrus and medially by the genual and cingulate gyri.

The gyrus is broader than the precruciate gyrus and was symmetrical in all examined hemispheres.

#### 3.2.3 Medial gyri

##### 3.2.3.1 Genual gyrus

The genual gyrus occupies the rostral medial surface, surrounding the genu of the corpus callosum.

It is bounded dorsally by the genual sulcus, ventrally by the endogenual and ectogenual sulci, and caudoventrally by the callosal sulcus. The gyrus is moderately broad, slightly convex, and continuous with the cingulate gyrus caudally (Figure 8). Its surface is smooth, with no major interruptions observed between hemispheres.

##### 3.2.3.2 Cingulate gyrus

The cingulate gyrus extends along the medial surface, following the course of the callosal sulcus.

It is bounded ventrally by the callosal sulcus and dorsally by the cingulate sulcus, extending rostrally from the genual gyrus to near the splenium caudally. The gyrus is elongated, gently arched, and uniformly broad along most of its length (Figure 8).

##### 3.2.3.3 Splenial gyrus

The splenial gyrus is located caudally around the splenium of the corpus callosum. It is bounded dorsocaudally by the splenial sulcus, medially by the caudal end of the callosal sulcus, and laterally by the ectosplenial sulcus. The gyrus is relatively narrow, elongated, and curves gently around the caudal end of the corpus callosum (Figure 8).

##### 3.2.3.4 Ectosplenial gyrus

The ectosplenial gyrus is positioned on the caudomedial surface of the hemisphere, lying dorsal and caudal to the splenial gyrus. It is bounded ventromedially by the splenial sulcus, dorsally by the ectosplenial sulcus, and dorsally by the marginal gyrus. The gyrus is elongated and follows an oblique rostrocaudal course. Its surface is moderately convex, with subtle variations in width along its length (Figure 8).

Across all specimens, the observed anatomical variation was confined to secondary morphological features and did not alter the overall cortical organization or the identification of the principal anatomical landmarks. Minor variations were most commonly observed within the suprasylvian and ectosylvian complexes, where differences in branching pattern and segment length were present. Less frequent variation involved the continuity of the endomarginal sulcus and minor hemispheric asymmetry in the marginal and coronal sulci. Importantly, no major sulci or gyri were absent in any specimen.

## 4 Discussion

The present study provides a comprehensive anatomical reference for the external cortical organization of the equine brain based on multiple adult specimens and standardized NAV terminology. The findings confirm previous observations that the horse possesses one of the most highly gyrencephalic cerebral cortices among domestic mammals (1, 4), while demonstrating that the principal cortical landmarks can be consistently identified using a unified nomenclature. By integrating detailed multiview illustrations with standardized anatomical terminology, this atlas complements existing equine neuroanatomical resources and provides an additional practical reference for comparative neuroanatomy and veterinary education.

### 4.1 Comparative context and nomenclature harmonization

The equine cerebral cortex exhibits a high degree of folding compared with that of other domestic ungulates and carnivores (20–23). In horses, the continuity of the ectosylvian, suprasylvian, coronal, and presylvian sulci is a distinctive feature, evident even during fetal development (4, 24), and contrasts with the fragmented sulci observed in ruminants (11, 12, 25).

Comparative studies further reinforce the uniqueness of the equine cortex. Lang et al. (4) reported that the equine cerebral cortex is significantly more complex than that of carnivores and that the gyration pattern in horses differs from the bow-like configuration typical of carnivores, displaying numerous secondary sulci instead (4, 22, 23).

Even among artiodactyls, the brains of the dromedary and Bactrian camels exhibit sulcal patterns that differ from those of other ungulates, with additional short accessory sulci that further complicate the convolutions (14, 26). Detailed neuroanatomical and vascular investigations of the dromedary camel further demonstrate its unique cerebral organization and the complexities of neurotopography across species (27, 28).

Studies on dromedary camels have also shown that sulci vary not only between species but also between hemispheres of the same individual, and that homologies and terminology across domestic mammals still require refinement (14, 15). The considerable inter- and intra-species variation underscores the need for species-specific atlases.

In our study, comparisons with other equine investigations highlight both the consistencies and the clarifications provided by our atlas.

Schmidt et al. (3) described the prorean, presylvian, diagonal, Sylvian, ectosylvian, suprasylvian, and oblique sulci, consistent with our observations, but did not report the accessory Sylvian fissure, which we consistently identified.

Böhme (29) mentioned the Sylvian and accessory Sylvian fissures along with the prorean, presylvian, and suprasylvian sulci, but omitted the ectosylvian sulcus, which our findings confirm as a constant feature.

Similarly, Lang et al. (4), who followed NAV standards, noted the presylvian, suprasylvian, Sylvian, and accessory Sylvian fissures but did not include the ectosylvian sulcus in the lateral view. The systematic inclusion of the ectosylvian sulcus in our study, as well as in Schmidt et al. (3), resolves this inconsistency and emphasizes its role as a stable landmark in the equine lateral cortex.

Our identification of the oblique sulcus on the lateral surface also aligns with Schmidt et al. (3).

Likewise, our recognition of an accessory Sylvian fissure corroborates Böhme (29) and Lang et al. (4) but extends beyond Schmidt et al. (3) description.

Collectively, these comparisons demonstrate that our atlas consolidates features previously described by different authors while presenting them within a standardized NAV-based framework. Among ruminants, calves and adult cattle exhibit deep and variable sulci, particularly along the splenial and ansate regions (11, 12). However, the presylvian sulcus in cattle and buffalo runs ventrally parallel to the rhinal fissure and often terminates blindly, lacking a homologous prorean gyrus (11, 13).

In contrast, horses consistently show a distinct prorean gyrus bounded by the presylvian sulcus.

The giraffe maintains an ungulate-type sulcal map but shows subtle remapping of the suprasylvian and splenial regions, highlighting evolutionary divergence among large-bodied herbivores (30).

In humans, the lateral (Sylvian) fissure is deeply developed and associated with the insula (31). In contrast, in horses and ruminants, the insular depression is relatively shallow (11). These differences underscore the importance of rigorous adherence to NAV terminology, which prevents confusion with anthropocentric terms such as “lateral fissure” versus “Sylvian fissure.”

Our atlas follows NAV terminology and presents previously reported labels, such as fissura pseudosylvia and Sylvian fissure, within a standardized anatomical framework. Likewise, differences in terminology reported in earlier publications, such as Bradley’s designation of the ectosylvian sulcus as suprasylvian (32), are discussed in relation to current NAV terminology. This harmonization facilitates cross-species comparison and enhances clinical relevance.

From a comparative neurobiological perspective, the extensive gyrification of the equine cerebral cortex likely reflects evolutionary adaptations that increase cortical surface area while maintaining a compact cranial volume. Although the present study did not investigate cortical function or development, the conserved organization of the principal sulci and gyri provides an anatomical framework for future studies exploring the developmental mechanisms underlying cortical folding and their relationship to functional specialization. In this context, standardized descriptions of cortical morphology facilitate meaningful comparisons across mammalian species and contribute to a better understanding of the evolutionary diversification of the cerebral cortex.

### 4.2 Dorsal surface comparisons

The overall organization of the dorsal cortical surface observed in the present study closely agrees with previous descriptions of the equine brain (3, 4), supporting the reproducibility of these principal anatomical landmarks across independent studies. In particular, the marginal complex represents a stable framework for describing the dorsal cortical surface, whereas the detailed documentation of the endomarginal sulcus helps clarify a feature that has received comparatively little attention in earlier reports. These observations contribute to a more complete and standardized description of equine dorsal cortical topography.

### 4.3 Medial surface comparisons

The medial cortical organization documented in the present study generally agrees with previous descriptions of the equine brain (3, 33), while providing additional clarification of several anatomical features. Schmidt et al. (3) identified the genual, endogenual, cingular, and splenial complex (ectosplenial, splenial, and suprasplenial sulci) but did not report the ectogenual sulcus. Oto and Hazıroğlu (33) also described the genual and cingular sulci but recognized only a single splenial sulcus, whereas, in our study, the recognition of distinct splenial, endosplenial, and ectosplenial sulci allows more precise delineation of the splenial region and highlights the value of standardized NAV terminology for resolving inconsistencies among earlier reports.

Likewise, the consistent identification of the accessory Sylvian fissure supports previous anatomical descriptions (4, 29) and reinforces its usefulness as a recognizable anatomical landmark within the equine cerebral cortex.

### 4.4 Relationship to previous equine atlases

Several previous studies have contributed substantially to the understanding of equine cerebral anatomy. Schmidt et al. (3) described equine neuroanatomy using high-field MRI, while Johnson et al. (2) developed a stereotaxic population-based atlas intended primarily for neuroimaging and neurosurgical applications. Lang et al. (4) revisited the cartographic organization of the equine neopallium and provided an important synthesis of cortical surface anatomy.

Unlike previous studies that primarily emphasized MRI visualization, stereotaxic mapping, or selected aspects of cortical morphology, the present study complements these contributions by integrating detailed documentation of the lateral, dorsal, rostral, and medial surfaces within a unified reference framework. Its principal contribution is the consolidation of external cortical anatomy using standardized NAV terminology and consistent multiview illustrations, providing a practical anatomical reference for comparative neuroanatomy and veterinary education. The use of standardized color coding further enhances the atlas by facilitating visual differentiation of adjacent sulci and gyri, improving anatomical orientation across multiple views, and providing a consistent visual framework that may support teaching and the interpretation of cortical surface anatomy.

### 4.5 Inter- and intra-individual variation

Although all principal sulci and gyri were identified in every specimen, minor variation was observed primarily in sulcal depth, branching patterns, and the length of individual sulcal segments, particularly within the suprasylvian and ectosylvian complexes, consistent with previous equine observations (4).

Previous reports describing marked variability in the cortical organization of ungulates have generally emphasized differences in secondary sulci, accessory branches, and variations in sulcal continuity or depth rather than the absence of the principal cortical landmarks (4, 11).

These findings suggest that, despite normal individual variation in cortical folding, the major anatomical landmarks remain sufficiently reproducible to support standardized anatomical description. Therefore, our observations are consistent with the available literature and indicate that variability in equine cortical morphology primarily affects secondary features, whereas the principal sulci and gyri remain reliably identifiable. Similar variability has been reported in ruminants (11), indicating that differences in secondary sulcal morphology are a common feature of ungulate brains rather than a species-specific characteristic. No systematic hemispheric asymmetry was observed in the present study, although subtle volumetric laterality has previously been reported in horses (2).

The limitations of this study include several factors that define its current scope. First, although the study included both sexes (4 males and 6 females) and adult horses aged approximately 7–10 years, the relatively small sample size precluded meaningful analysis of sex, age, and breed-related differences in cortical morphology. Therefore, our atlas represents the most common topography observed in adult mixed-breed horses but cannot account for potential population-level variation in gyrification patterns. Second, this work is focused on gross topographic mapping and nomenclature standardization and intentionally lacks quantitative morphometric assessments, such as the gyrification index or precise sulcal depth measurements. While this aligns with the atlas’s primary goal of resolving surface architecture, future studies should integrate these quantitative metrics to provide a full three-dimensional characterization of the equine neopallium. In addition, all specimens were fixed in formaldehyde for one month prior to brain extraction to ensure uniform preservation. Although prolonged fixation may result in some degree of tissue shrinkage, all brains underwent the same fixation protocol, thereby minimizing its influence on the comparative anatomical observations presented in this study. Future work can address these limitations by incorporating comprehensive morphometric analyses across diverse equine populations and integrating three-dimensional reconstructions with *in vivo* imaging templates, thereby expanding the anatomical utility of the atlas and providing a foundation for future translational investigations.

Developmental and breed-based investigations, along with efforts to establish cross-species consensus in cortical nomenclature, will further refine the comparative and translational value of equine neuroanatomical atlases.

Furthermore, the present atlas complements recent studies describing the vertebrobasilar and carotid systems contributing to the cerebral arterial circle of the horse, collectively expanding the anatomical resources available for equine neuroscience, neuroimaging, and clinical investigations (34, 35).

## 5 Conclusion

Complementing previous MRI-based, stereotaxic, and anatomical descriptions, the present work provides integrated multiview documentation of the external cerebral architecture using standardized NAV terminology. By systematically documenting the lateral, dorsal, rostral, and medial surfaces of multiple adult specimens, we provide a detailed reference for equine cortical sulci and gyri, using standardized NAV terminology to consolidate previous anatomical descriptions within a unified framework. This work synthesizes existing knowledge of the equine cerebral cortex and complements recent atlases of other domestic mammalian species. Its principal contribution is to provide a standardized, specimen-based anatomical reference for comparative neuroanatomy and veterinary education. The atlas may also serve as an anatomical resource for neuroimaging interpretation and anatomical orientation, and provide a framework for future morphometric, developmental, and functional investigations.

## Data Availability

The original contributions presented in the study are included in the article/supplementary material, further inquiries can be directed to the corresponding author.
